# Short- and long-term reproducibility of diffusion-weighted magnetic resonance imaging of lower extremity musculature in asymptomatic individuals and a comparison to individuals with spinal cord injury

**DOI:** 10.1186/s12891-018-2361-7

**Published:** 2018-12-06

**Authors:** Jacob G. McPherson, Andrew C. Smith, Daniel A. Duben, Katie L. McMahon, Marie Wasielewski, Todd B. Parrish, James M. Elliott

**Affiliations:** 1 Department of Biomedical Engineering, Florida International University, Miami, FL USA; 2 Department of Physical Therapy and Human Movement Sciences, Northwestern University Feinberg School of Medicine, Chicago, IL USA; 3 School of Physical Therapy, Regis University, Denver, CO USA; 4 Herston Imaging Research Facility, University of Queensland Centre for Clinical Research, Herston, QLD Australia; 5 School of Clinical Sciences, Institute of Health and Biosciences Innovation, Queensland University of Technology, Brisbane, Australia; 6 Department of Radiology, Northwestern University, Chicago, IL USA; 7 School of Health and Rehabilitation Sciences, The University of Queensland, Brisbane, Australia; 8 Faculty of Health Sciences, The University of Sydney, Northern Sydney Local Health District, St Leonards, NSW Australia

**Keywords:** Magnetic resonance imaging, Skeletal muscle, Spinal cord injury, Diffusion-weighted imaging

## Abstract

**Background:**

Diffusion-weighted magnetic resonance imaging (DW-MRI) of skeletal muscle has the potential to be a sensitive diagnostic and/or prognostic tool in complex, enigmatic neuromusculoskeletal conditions such as spinal cord injury and whiplash associated disorder. However, the reliability and reproducibility of clinically accessible DW-MRI parameters in skeletal muscle remains incompletely characterized – even in individuals without neuromusculoskeletal injury – and these parameters have yet to be characterized for many clinical populations. Here, we provide normative measures of the apparent diffusion coefficient (ADC) in healthy muscles of the lower limb; assess the rater-based reliability and short- and long-term reproducibility of the ADC in the same muscles; and quantify ADC of these muscles in individuals with motor incomplete spinal cord injury.

**Methods:**

Twenty individuals without neuromusculoskeletal injury and 14 individuals with motor incomplete spinal cord injury (SCI) participated in this investigation. We acquired bilateral diffusion-weighted MRI of the lower limb musculature in all participants at 3 T using a multi-shot echo-planar imaging sequence with b-values of 0, 100, 300 and 500 s/mm^2^ and diffusion-probing gradients applied in 3 orthogonal directions. Outcome measures included: (1) average ADC in the lateral and medial gastrocnemius, tibialis anterior, and soleus of individuals without neurological or musculoskeletal injury; (2) intra- and inter-rater reliability, as well as short and long-term reproducibility of the ADC; and (3) estimation of average muscle ADC in individuals with SCI.

**Results:**

Intra- and inter-rater reliability of the ADC averaged 0.89 and 0.79, respectively, across muscles. Least significant change, a measure of temporal reproducibility, was 4.50 and 11.98% for short (same day) and long (9-month) inter-scan intervals, respectively. Average ADC was significantly elevated across muscles in individuals with SCI compared to individuals without neurological or musculoskeletal injury (1.655 vs. 1.615 mm^2^/s, respectively).

**Conclusions:**

These findings provide a foundation for future studies that track longitudinal changes in skeletal muscle ADC of the lower extremity and/or investigate the mechanisms underlying ADC changes in cases of known or suspected pathology.

## Background

Diffusion-weighted magnetic resonance imaging (DW-MRI) provides a potential diagnostic and prognostic measure for the normal (and abnormal) movement of water through living soft-tissues. Skeletal muscle is an emerging application for DW-MRI [1], where it holds considerable potential in common, yet enigmatic, clinical conditions. For example, significant changes in DW-MRI metrics have been observed in individuals with rotator cuff tears [2], muscular dystrophy [3], myopathies [4, 5], radiculopathy [6, 7], and in animal models of denervation [7, 8]. In cases, diffusion-related changes may also precede findings on other diagnostic tests [7], opening a critical early window where diagnostic and prognostic data could better inform intervention. Additionally, DW-MRI could serve as a marker of muscle response to therapeutic exercise [9, 10]. Despite the increasing number of investigations utilizing DW-MRI in skeletal muscle – and in particular the use of skeletal muscle DW-MRI as a diagnostic or outcome measure – normative data remains sparse. Complicating matters further, the rater-based reliability and temporal reproducibility of DW-MRI-related parameters have also yet to be established for skeletal muscle.

Here, we investigate a clinically relevant DW-MRI parameter, the Apparent Diffusion Coefficient (ADC), in muscles of the lower limb. We estimate the rater-based reliability and the temporal reproducibility of ADC at both short- and long-inter-scan intervals, with the primary goal of providing an additional statistical reference for future investigations incorporating DW-MRI of skeletal muscle. We also quantify the ADC both in a cohort of individuals without neurological or musculoskeletal injury and in a cohort of individuals with motor incomplete spinal cord injury, a potential target for clinical use of DW-MRI. We hypothesize that ADC will be elevated in leg muscles innervated by spinal nerves below the level of lesion, given the previously documented elevations in ADC following denervation and/or trauma [2, 7].

## Methods

### Study participants

In total, 20 individuals without known neurological or musculoskeletal injury (“control cohort”; 11 males, 9 females; age: 31 ± 10 yrs) and 14 individuals with motor incomplete spinal cord injury (“SCI cohort”; 13 males, 1 female; age: 43 ± 12 yrs.; Table 1; see also [11]) participated in this investigation. All individuals provided informed, written consent to participate, and the protocol was approved by the Institutional Review Board of Northwestern University in accordance with the guidelines established by the Declaration of Helsinki for research involving human participants.

**Table 1:** Spinal cord injury cohort demographic and clinical data

Participant	Gender	Age	Time since injury (years)	Level of injury	ASIA Impairment Scale	Primary ambulation
1	M	53	6	C5	C	Wheelchair
2	M	57	8	C3	D	Walk with A.D.
3	F	52	0.5	C6	D	Walk with A.D.
4	M	31	4	C5	D	Walk with A.D.
5	M	28	3	C5	D	Walk
6	M	50	2	C6	D	Walk
7	M	30	4	C6	C	Wheelchair
8	M	27	4	C5	C	Wheelchair
9	M	32	5	C7	D	Walk
10	M	45	3.5	C3	D	Walk with A.D.
11	M	36	1.5	C6	C	Wheelchair
12	M	50	5	C4	D	Wheelchair
13	M	64	4	C5	D	Wheelchair
14	M	52	31	C5	D	Wheelchair

*Cx* cervical spinal segment number, *A.D.* assistive device

Participants with any of the following were excluded from participation: previous diagnosis of cervical or lumbar radiculopathy; history of neurological disorders (e.g. Multiple Sclerosis, previous stroke, myelopathy), inflammatory diseases (e.g. Hepatitis, Systemic Lupus Erythematosus, Rheumatoid Arthritis or Osteoarthritis, Alzheimers, Ankylosing Spondylitis, Chron’s Disease, Fibromyalgia) or metabolic disorders (e.g. Diabetes, hyper- and hypo-thyroidism); previous spinal fracture or spinal surgery and/or history of one or more motor vehicle collisions (neither criteria applied to participants in the SCI cohort); and standard/institutional contraindications to MRI, including claustrophobia, metallic implants, pacemaker and pregnancy or thought to be pregnant in the absence of contraception since the last normal menstrual period.

Average ADC per muscle was estimated in all participants (control: *N* = 20; SCI: *N =* 14). For the control cohort, we also quantified the rater-based reliability, short-term reproducibility, and long-term reproducibility of the ADC in subsets of these individuals: intra- and inter-rater reliability were estimated from 6 individuals (3 male, 3 female), short-term reproducibility was estimated in 3 of these 6 (2 male, 1 female), and long-term reproducibility was estimated in a separate subset of 12 individuals (8 males, 4 females). Reliability metrics were not computed for the SCI cohort because it would not have been feasible to decouple ADC changes related to evolution of the post-SCI recovery/impairment sequelae from variability inherent to the DW-MRI technique itself at this early, foundational stage of investigation.

### DW-MRI acquisition and analysis

MRI scans were performed on a Siemens 3 T TRIO MRI scanner (Siemens; Erlangen, Germany). A 16-channel body array coil positioned anteriorly over the lower legs. Segments 3 and 4 of the spine matrix coil were also turned on to improve signal posteriorly. The legs were comfortably stabilized (to minimize movement) using sandbags. Axial diffusion-weighted images (DWI) were acquired bilaterally using a multi-shot echo-planar imaging sequence (readout segmentation of long variable echo-trains; RESOLVE [12]) with the following parameters: repetition time: 2500 ms; echo time 1: 67 ms; echo time 2: 113 ms; matrix: 224 × 224; field of view: 320 mm; echo spacing: 0.34 ms; bandwidth: 531 Hz/Px; number of averages: 1; slice thickness: 5 mm with a gap spacing of 5 mm; total scan time: 4:39 mins. Fat suppression was used to eliminate signal from subcutaneous fat. Diffusion-probing gradients were applied in 3 orthogonal directions (*x, y,* and *z)* at the following levels of diffusion weighting (b-values): 0, 100, 300 and 500 s/mm^2^, where b_0_ reflects no diffusion weighting (i.e., a T2-weighted image). Isotropic DWI’s were calculated automatically at each b-value as part of the image reconstruction (Siemens VB17A software).

**Fig. 1:**
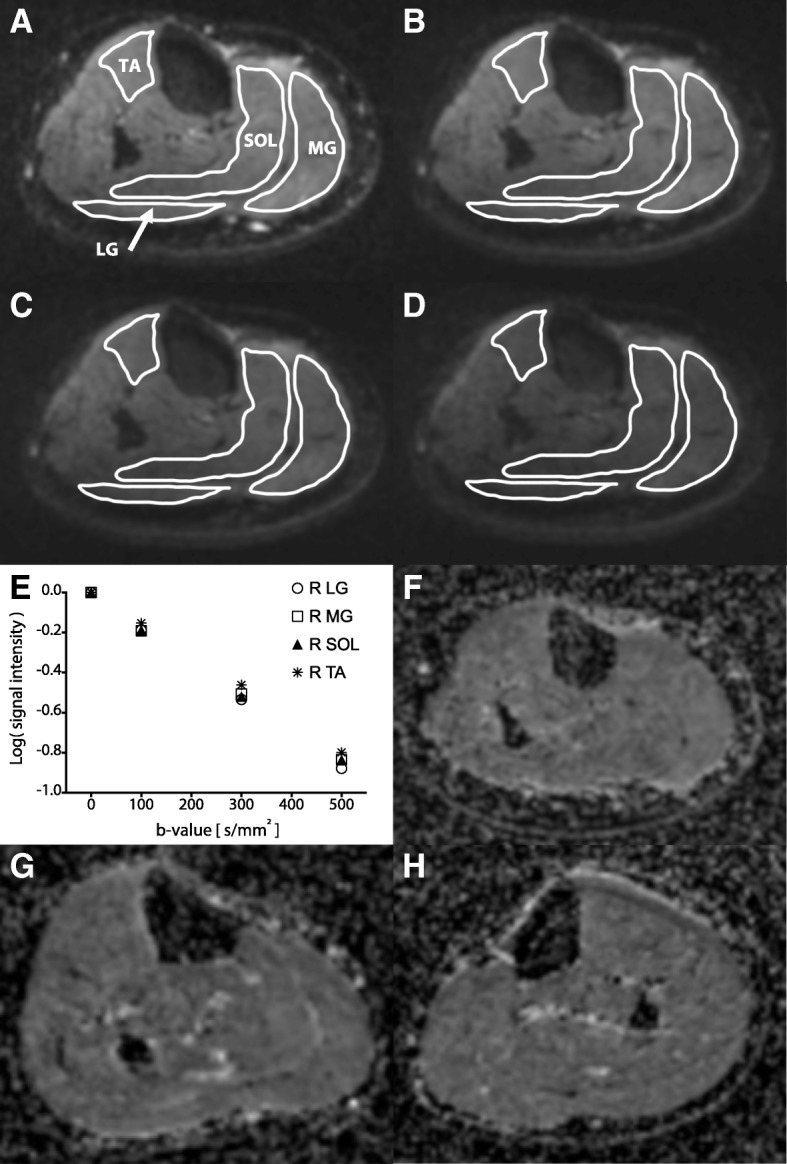
Representative DW-MRI images. All images acquired at the same proximal-distal level, approximately halfway between the proximal and distal tibial epiphyses, in the right leg of a single, representative participant. **a**-**d** Diffusion-weighted images acquired at progressively increasing diffusion-probing gradient strength: (**a**) b = 0 s/mm^2^, (**b**) b = 100 s/mm^2^, (**c**) b = 300 s/mm^2^, and (**d**) b = 500 s/mm^2^. **e** logarithm of signal intensity is plotted as a function of diffusion-probing gradient strength in a single participant. **f** ADC map for the same participant at (**a**-**e**). **g**, **h** ADC maps for the right and left legs of a representative participant with spinal cord injury. Abbreviations: TA, Tibialis Anterior; SOL, Soleus; LG, Lateral Gastrocnemius; MG, Medial Gastrocnemius

Ten equidistant image slices were acquired between the proximal tibial epiphysis and the distal tibial epiphysis at each b-value. In each slice, regions of interest (ROI) were defined bilaterally using the native axial T1 image as a guide, then transferred to the corresponding isotropic DWI at each successive b value. Blinded, novice raters with ≤1-year experience manually produced all ROI’s. ROIs delineated the tibialis anterior (TA), soleus (SOL), and lateral and medial gastrocnemius (LG, MG, respectively). ROI were identified using Analyze 11.0 (Biomedical Imaging Resource, Mayo Clinic, Rochester, MN) and OsiriX 7.5.1 (Pixmeo SARL, Bernex, Switzerland), with the perimeter of each ROI defined as the fascial plane of each muscle at a given proximal-distal location (Fig. 1a).

For a given ROI and b-value, we extracted the mean pixel intensity per DWI and averaged across slices to arrive at the grand mean throughout the muscle’s proximal-distal extent. This resulted in 4 discrete estimates of signal intensity per muscle (i.e., one for each b-value), as seen in Fig. 1a-d. From these values, we computed the apparent diffusion coefficient (ADC) for each muscle, using regression analysis to fit pixel intensity and b-value to the standard mono-exponential diffusion equation: SI_*b*_ = SI_0_ × *e*^(−b*ADC)^ (Fig. 1e). In this equation, SI_*b*_ is signal intensity at a particular non-zero b-value and SI_0_ is signal intensity at b = 0 s/mm^2^. Figure 1f shows a pixel-wise ADC map for a single representative participant without known neurological or musculoskeletal injury; Fig. 1g, h depict ADC maps for the right and left legs of a participant with spinal cord injury. All statistical calculations described below are based upon whole muscle ADC estimates.

To estimate short-term reproducibility, we acquired two DW-MRI scans approximately 30 min apart. Between scans, participants fully exited the MRI scanner environment and waited in a temperature-controlled room (72.6 ° F); activity level and fluid intake were kept to a minimum during this interval. To estimate long-term reproducibility, we acquired two DW-MRI scans approximately 9 months apart. We did not control for activity level, hydration, or time of day between long-term reliability scans.

### Statistical analyses

We used both relative and absolute estimates of measurement error to quantify the reliability and reproducibility of skeletal muscle ADC. Relative estimates of measurement error were based on intra-class correlation coefficients (ICC_2,1_; two-way random, absolute agreement), and were used to compute intra- and inter-rater reliability of single scans, as well as short- and long-term ADC reproducibility. For inter-rater reliability, 4 raters independently defined each ROI. Two of these raters also performed duplicate ratings on the same images, and these data were used to compute intra-rater reliability. Two additional raters defined all ROI used to compute estimates of short- and long-term reproducibility.

Absolute measures of reliability and reproducibility, also known as precision statistics, included root-mean-square standard deviation (RMS-SD), root-mean-square coefficient of variation (RMS-CV), and least significant change (LSC), expressed at the 95% confidence level. For reference, LSC is based upon the Repeatability Coefficient originally defined by Bland and Altman [13]. The formula for RMS-SD is as follows,

$$ RMS- SD\ \left(\times 10{e}^{-3}\ \frac{mm^2}{s}\right)=\sqrt{\frac{\kern0.75em {\Sigma}_{i=1}^N\left({\varSigma}_{r=1}^R\frac{{\left(\widehat{ADC_r}-\overline{ADC}\right)}^2}{R}\right)}{N}} $$

where *r* is an individual rating of a given image (for intra-rater reliability) or a single rater (for inter-rater reliability), *R* is the total number of individual ratings of a given image (for intra-rater reliability) or the total number of raters (for inter-rater reliability), *i* is a single image of a given muscle, and *N* is the total number of images rated across all study participants in a given comparison (i.e., number of images *i* multiplied by the number of participants). The formulae for RMS-CV and LSC follow from RMS-SD, and are:

$$ RMS- CV\ \left(\%\right)=\left(\frac{RMS- SD}{grand\ mean\ (ADC)}\right)\times 100 $$

$$ LSC\ \left(\%\right)=\left( RMS- CV\right)\times 2.77 $$

Absolute measures of error were computed for rater-based reliability as well as short- and long-term reproducibility.

We used a linear mixed model to assess potential differences in ADC between individuals without neuromuscular injury and individuals with SCI. The model tested the main fixed effects of cohort (i.e., control vs. SCI) and muscle on average ADC, as well as the cohort-by-muscle interaction and the effect of ambulation type (i.e., walking vs. wheelchair) within the SCI cohort. Participant was included in the model as a correlated random effect. Nominal sample size was determined a priori to be 13 individuals per cohort. Sample size estimates were based on the following model parameters: a small standardized effect size (0.2), α error probability of 0.05, power (1-β error probability) of 0.85, 2 groups (control and SCI), 4 repeated measures (i.e., 4 muscles), and a moderate correlation among repeated measures (0.7). All statistical comparisons were considered significant at the α = 0.05 level. All statistical analyses were performed with IBM SPSS Statistics software (Version 19; SPSS Inc., Armonk, NY, USA).

## Results

### ADC by muscle

Table 2 summarizes the average ADC per muscle across the control and SCI cohorts. For the SCI cohort, we also computed the average ADC per muscle when the legs were grouped by residual strength rather than left/right.

**Table 2:** Mean ADC by muscle (× 10^− 3^ mm^2^/s)

	Control L	Control R	SCI L	SCI R	SCI more impaired	SCI less impaired
MG	1.62 ± 0.08	1.63 ± 0.13	1.66 ± 0.12	1.65 ± 0.10	1.65 ± 0.11	1.67 ± 0.12
LG	1.62 ± 0.12	1.60 ± 0.17	1.69 ± 0.14	1.70 ± 0.08	1.67 ± 0.08	1.72 ± 0.13
TA	1.61 ± 0.08	1.59 ± 0.10	1.61 ± 0.11	1.58 ± 0.09	1.59 ± 0.09	1.59 ± 0.10
SOL	1.63 ± 0.09	1.63 ± 0.09	1.69 ± 0.12	1.66 ± 0.10	1.66 ± 0.10	1.69 ± 0.12
Average	1.62 ± 0.09	1.61 ± 0.13	1.66 ± 0.04	1.65 ± 0.05	1.64 ± 0.10	1.67 ± 0.12

Data presented as mean ± standard deviation

### Rater-based reliability

Rater-based reliability statistics of the first time-point can be seen in Table 3. Data are presented as the mean of left and right legs, and excluded images are the same as those described above. ICC-based estimates are presented per muscle, and absolute measures of error are presented as averages across muscles. The LG and SOL were the most inconsistently defined muscles. For LG, errors appeared to stem from an inability to reliably distinguish the border between the LG and MG on the DW-MRI image; for SOL, errors appeared to stem from an inability to reliability distinguish the interior perimeter of the SOL from deeper muscles such as the flexor digitorum longus, flexor hallucis longus, and the tibialis posterior.

**Table 3:** Relative and absolute rater-based reliability

	MG (ICC)	LG (ICC)	TA (ICC)	SOL (ICC)	RMS-SD (×10^−3^ mm^2^/s)	RMS-CV (%)	LSC (%)
Intra-rater (R1)	1.00	0.90	0.91	0.60	0.06	3.53	9.77
Intra-rater (R2)	0.99	0.79	0.97	0.99	0.04	2.58	7.16
Inter-rater	0.81	0.72	0.87	0.76			

*Abbreviations ICC* intra-class correlation coefficient, *RMS-SD* root-mean-square standard deviation, *RMS-CV* root-mean-square coefficient of variation, *LSC* least significant change

### Short- and long-term reproducibility

Given the high rater-based reliability, we estimated short-term reproducibility in only one leg per participant. For these analyses, no data required removal due to artifacts. Short-term reproducibility, assessed by ICC_2,1_, was 0.94. Short-term RMS-SD averaged 0.03 × 10^− 3^ mm^2^/s across muscles and participants, with a mean RMS-CV of 1.63% and an LSC of 4.50%. For estimates of long-term reproducibility, we used all available data across both legs of the 11 participants (the left LG was removed in 8 participants due to artifacts, as was the left SOL in 1 participant and the MG bilaterally in 2 participants). With a 9-month inter-scan interval, we found an ICC-based reproducibility of 0.34. RMS-SD averaged 0.07 × 10^− 3^ mm^2^/s over this interval, with a mean RMS-CV of 4.33% and an LSC of 11.98%.

### ADC in chronic SCI

**Fig. 2:**
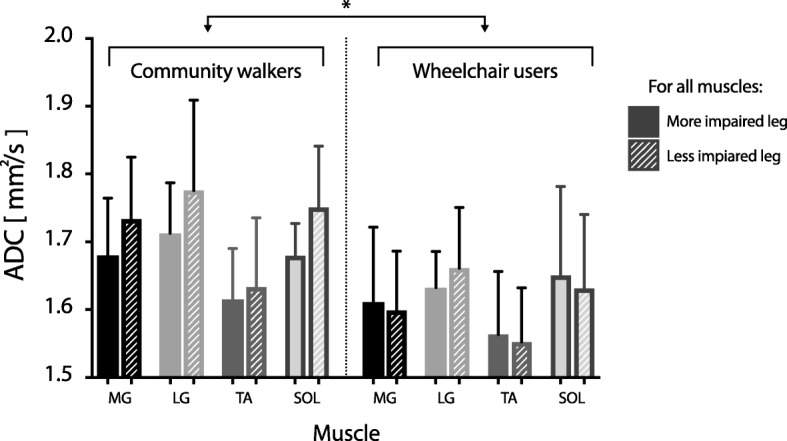
Average ADC is elevated in community walkers compared to wheelchair users post-SCI. Average ADC is shown per muscle for the SCI cohort, parsed by ambulation type (community walkers vs. wheelchair users) and grouped by overall impairment of each leg. Solid tone columns: more impaired leg; cross-hatched columns: less impaired leg. MG: medial gastrocnemius; LG: lateral gastrocnemius; TA, Tibialis Anterior; SOL, Soleus

We found a significant main effect of participant cohort on average ADC per muscle (*F* = 5.94; *P =* 0.016), reflective of an elevated average ADC across muscles in individuals with SCI compared to individuals without neuromuscular injury (Table 2). We also found a significant effect of ambulation type on average ADC within the SCI cohort, driven by a higher average ADC across muscles in community walkers than wheelchair users (*F* = 19.06; *P* < 0.001; Fig. 2). Neither the main effect of muscle (*F* = 1.77, *P =* 0.095) nor the muscle-by-cohort interaction (*F* = 1.47, *P =* 0.18) were significant at the α = 0.05 level. Qualitatively, the non-significant main effect of muscle appeared to be driven primarily by low ADC values for the TA muscle in the SCI cohort. We confirmed this observation by conducting a separate one-way repeated measures ANOVA on the SCI cohort alone; a within-subject factor of muscle and an independent variable of average ADC revealed a significant main effect of muscle on average ADC (*F* = 6.25; *P =* 0.002). Post-hoc analyses (after Bonferroni correction) confirmed that the effect of muscle was due to significantly lower average ADC in the TA compared to the MG, LG, and SOL (*P =* 0.004 and *P =* 0.0005 for L and R TA, respectively).

## Discussion

The objectives of this investigation were to provide normative ADC values in healthy skeletal muscle at 3 T, to further establish the rater-based reliability and temporal reproducibility of DW-MRI in muscle, and to estimate ADC in a cohort of individuals with motor incomplete spinal cord injury. Although previous reports of mean ADC for healthy skeletal muscles exhibit considerable variability, our estimates are generally consistent with the available data. In the earliest results, obtained at 0.5 T, Morvan et al. reported mean ADC of resting muscle at 1.82 × 10^− 3^ mm^2^/s [14]. ADC estimates obtained at 1.5 T have generally been lower than our results, ranging from 1.26 × 10^− 3^ mm^2^/s to 1.51 × 10^− 3^ mm^2^/s [7, 15, 16], although more recently, values of 1.8 × 10^− 3^ mm^2^/s have again been reported (albeit at the shoulder) [2]. ADC estimates of leg muscles obtained at 3 T appear to be more closely aligned with our findings, ranging from 1.51–1.85 × 10^− 3^ mm^2^/s [3–5].

Overall, we found excellent absolute rater-based reliability and short-term reproducibility for ADC in skeletal muscle. Our average rater-based LSC was less than 10% across muscles, and our average LSC for short-term reproducibility was 4.5% for scans acquired on the same day. Both findings are consistent with the available literature [17, 18]. Based upon standard reporting conventions for relative reliability metrics [19], our ICC estimates for rater-based reliability and short-term reproducibility were also excellent. We caution against direct comparisons of our ICC values to other studies however, given that ICC estimates are highly sensitive to sample size and across-participant variability not attributable to the instrument itself. For example, while Heemskerk et al. [18] report much lower ICCs of 0.7 and 0.17 for rater-based reliability and short-term reproducibility, their repeatability coefficients (i.e., LSC) for the same data were closely aligned with our results, at 6 and 12%, respectively.

Experimental and/or physiological factors likely contributed to our lower long-term ICC estimates. For example, our study implicitly assumed that ADC values in healthy skeletal muscle are stable over time in the absence of trauma, disease, etc. This is a clear oversimplification, as changes in temperature, activity level, and hydration are all capable of influencing the ADC of skeletal muscles [9, 10, 15, 20]. Because we did not explicitly control for these factors, our slightly lower long-term performance is presumably related, at least in part, to true changes in muscle physiology that were unaccounted for by our experimental design. Nevertheless, our estimate of absolute long-term reproducibility is consistent with those reported in the literature, which range from repeatability coefficients of 7–15% with scans acquired 1–2 weeks apart [18, 21].

We found a modest but statistically significant increase in average ADC across muscles in individuals with motor incomplete SCI compared to individuals without neurological or musculoskeletal injury. These increases were confined to the plantarflexor muscles (MG, LG, and SOL), with the TA showing no clear differences between cohorts. Given that elevations of muscle ADC, not reductions, are most commonly associated with pathology, it was somewhat surprising that average ADC was higher in individuals with SCI for whom walking is the primary means of ambulation compared to wheelchair users. It should be reiterated however that neural drive to leg muscles was not altogether absent in our wheelchair user cohort; these participants could perform standing transfers and engage in some therapist assisted walking. Nevertheless, changes in ADC both within the SCI cohort and between the SCI and control cohorts presumably reflect a combination of direct, SCI-induced alterations in neural drive to muscles and indirect effects associated with reduced motor output. Indeed, denervation, atrophy, and changes in physical activity level are all known to influence skeletal muscle ADC [7, 8, 10, 22].

Because our study was not intended to correlate ADC with motor function, the relevance of elevated ADC to impairment – if any – remains unclear. Nevertheless, it is interesting to note that plantarflexor spasms represent one of the most common presentations of spasticity after SCI [23], and we observed preferential elevation of ADC in these muscles. Future work that explores the mechanisms of ADC elevation post-SCI and their potential relevance to motor function, as well as normative estimates of ADC in motor complete SCI, is clearly warranted.

Our study has important limitations. Unlike previous investigations, our ROI definition was performed by raters with minimal imaging experience yet an intimate knowledge of the relevant anatomical structures (Doctor of Physical Therapy students). Although this choice was motivated by a desire to estimate a potential lower bound on rater-based reliability, it also complicates the interpretation of our findings by making it harder to decouple the sources of experimental, physiological, and rater-induced variability. Another limitation is that we used the same MRI scanner for all imaging sessions. As a result, our ADC values, as well as our reliability estimates, should be interpreted only in this context. In purest terms, our results may over-estimate rater-based reliability and/or reproducibility and thus be much different if compared to results from multi-site investigations incorporating different community imaging centers. Finally, we did not control leg temperature in the cohort of individuals with SCI. Thus, it is possible that subtle temperature differences existed between the SCI and control cohorts and/or between the more and less impaired legs of individuals with SCI. Reduced temperature in the extremities, as has been reported in SCI [24], could change our estimates of ADC independently of SCI-induced alterations of muscle physiology [15, 20]. It seems unlikely that changes in temperature could explain the differences in ADC we found between individuals with and without SCI, however. Specifically, leg temperature in individuals with SCI is generally lower than that of individuals without SCI [24], suggesting that ADC should be lower in SCI than controls [20]. However, we found a significantly *elevated* ADC across muscles in the SCI cohort compared to the control cohort, indicating that temperature was not the primary factor driving the observed ADC differences. We also found no significant differences in ADC between legs within the SCI cohort for any muscle (Fig. 2). This finding also suggests that the influence of leg temperature on our results was likely minimal (although it was presumably a source of overall variability in our estimates of ADC). Nevertheless, future work investigating skeletal muscle ADC in individuals with SCI (or other neurological injuries) should systematically monitor and control extremity temperature to avoid undue experimental confounds.

## Conclusions

Despite the promise of DW-MRI in skeletal muscle as an additional tool for clinical evaluation and basic scientific investigation, its utility has remained limited at least in part due to a lack of normative data and an incomplete assessment of its precision and reliability. Our results demonstrate that its reproducibility is excellent over short time scales, reinforcing the available literature. We also extend these findings by providing insights into the stability of the approach over the longest inter-scan interval available to date (9 months). Although performance was expectedly lower over this time scale, ADC changes as small as 12% were still discernable. Considering that ADC changes of ~ 24% have been reported in radiculopathy [7], ~ 35% following rotator cuff tear [2], and ~ 20% immediately following vigorous contraction of the same muscles included in this investigation [10], we conclude that controlled experiments using DW-MRI of skeletal muscle are sufficiently reproducible for longitudinal analyses. With evolving scanner and sequence technologies, DW-MRI may prove to be a highly sensitive measure of physiological changes to peripheral muscles after neurological injury and in any number of common, yet enigmatic, neuro-musculoskeletal conditions (e.g., low back pain, whiplash). Such knowledge could help to improve clinical decision making at a time when therapeutic intervention may have its largest effect.

## Abbreviations

3T: 3-Tesla
ADC: Apparent diffusion coefficient
DW-MRI: Diffusion-weighted magnetic resonance imaging
ICC: Intra-class correlation coefficient
LG: Lateral gastrocnemius
LSC: Least significant change
MG: Medial gastrocnemius
MRI: Magnetic resonance imaging
RMS-CV: Root-mean-square coefficient of variation
RMS-SD: Root-mean-square standard deviation
ROI: Region of interest
SCI: Spinal cord injury
SOL: Soleus
TA: Tibialis anterior
